# Occurrence, antimicrobial susceptibility, and resistance genes of *Staphylococcus aureus* in milk and milk products in the Arsi highlands of Ethiopia

**DOI:** 10.1186/s12866-024-03288-3

**Published:** 2024-04-16

**Authors:** Abiot Deddefo, Gezahegne Mamo, Minda Asfaw, Adem Edao, Adem Hiko, Dereje Fufa, Mohammed Jafer, Melaku Sombo, Kebede Amenu

**Affiliations:** 1 College of Agriculture and Environmental Sciences, Arsi University, P.O. Box 193, Asella, Ethiopia; 2 College of Veterinary Medicine and Agriculture, Addis Ababa University, P.O. Box 34, Bishoftu, Ethiopia; 3 College of Veterinary Medicine, Haramaya University, P.O. Box 138, Haramaya, Ethiopia; 4 Asella Regional Veterinary Laboratory, P.O. Box 212, Asella, Ethiopia; 5 National Animal Health Institute, P.O. Box 04, Sebeta, Ethiopia; 6 Animal and Human Health Programme, International Livestock Research Institute (ILRI), P.O. Box 5689, Addis Ababa, Ethiopia

**Keywords:** Antimicrobial resistance, Cottage cheese, Milk, *mecA* and *blaZ* genes, *Staphylococcus aureus*, Yogurt

## Abstract

**Background:**

In Ethiopia, milk production and handling practices often lack proper hygiene measures, leading to the potential contamination of milk and milk products with *Staphylococcus aureus* (*S. aureus*), including methicillin-resistant strains, posing significant public health concerns. This study aimed to investigate the occurrence, antimicrobial susceptibility profiles and presence of resistance genes in *S. aureus* strains isolated from milk and milk products.

**Methods:**

A cross-sectional study was conducted in the Arsi highlands, Oromia, Ethiopia from March 2022 to February 2023. A total of 503 milk and milk product samples were collected, comprising 259 raw milk, 219 cottage cheese, and 25 traditional yogurt samples. *S. aureus* isolation and coagulase-positive staphylococci enumeration were performed using Baird-Parker agar supplemented with tellurite and egg yolk. *S. aureus* was further characterized based on colony morphology, Gram stain, mannitol fermentation, catalase test, and coagulase test. Phenotypic antimicrobial resistance was assessed using the Kirby–Bauer disc diffusion method, while the polymerase chain reaction (PCR) was employed for confirming the presence of *S. aureus* and detecting antimicrobial resistance genes.

**Results:**

*S. aureus* was detected in 24.9% of the milk and milk products, with the highest occurrence in raw milk (40.9%), followed by yogurt (20%), and cottage cheese (6.4%). The geometric mean for coagulase-positive staphylococci counts in raw milk, yogurt, and cottage cheese was 4.6, 3.8, and 3.2 log_10_ CFU/mL, respectively. Antimicrobial resistance analysis revealed high levels of resistance to ampicillin (89.7%) and penicillin G (87.2%), with 71.8% of the isolates demonstrating multidrug resistance. Of the 16 *S. aureus* isolates analyzed using PCR, all were found to carry the *nuc* gene, with the *mecA* and *blaZ* genes detected in 50% of these isolates each.

**Conclusion:**

This study revealed the widespread distribution of *S. aureus* in milk and milk products in the Arsi highlands of Ethiopia. The isolates displayed high resistance to ampicillin and penicillin, with a concerning level of multidrug resistance. The detection of the *mecA* and *blaZ* genes in selected isolates is of particular concern, highlighting a potential public health hazard and posing a challenge to effective antimicrobial treatment. These findings highlight the urgent need to enhance hygiene standards in milk and milk product handling and promote the rational use of antimicrobial drugs. Provision of adequate training for all individuals involved in the dairy sector can help minimize contamination. These measures are crucial in addressing the threats posed by *S. aureus*, including methicillin-resistant strains, and ensuring the safety of milk and its products for consumers.

**Supplementary Information:**

The online version contains supplementary material available at 10.1186/s12866-024-03288-3.

## Introduction

Milk and milk products are rich in various nutrients, such as proteins, fats, carbohydrates, vitamins, and minerals, playing a crucial role in human nutrition [1]. On the other hand, they are frequently contaminated by various pathogens, including *Staphylococcus aureus* (*S. aureus*), which poses significant health risks to consumers [2]. *S. aureus* is a commensal and opportunistic pathogen that colonizes the skin, nasal passages, and mucosal membranes of both humans and animals. In dairy cattle, it is a major cause of mastitis, resulting in considerable economic losses [3]. In humans, *S. aureus* can lead to a spectrum of diseases, ranging from minor skin infections to severe conditions such as pneumonia and toxic shock syndrome [4]. Milk is an important source of staphylococcal food poisoning [5]. Several foodborne outbreaks of *S. aureus* intoxications have been documented to be associated with the consumption of contaminated milk [6–8]. Contamination of milk and milk products by *S. aureus* occurs throughout the entire production chain from milking to distribution. Factors such as substandard milking hygiene, contaminated udders, inadequate sanitation of milk equipment, and unclean environments, contribute to the entry of bacteria [9]. To effectively control *S. aureus* contamination in dairy products, it is crucial to implement rigorous hygiene protocols, conduct regular monitoring, and strictly adhere to food safety standards [9–11].

Antibiotics play a crucial role in treating bacterial infections in both humans and animals. However, their overuse and misuse can lead to the emergence and spread of antibiotic-resistant strains [12]. This presents a serious public health concern, limiting treatment options and increasing healthcare costs for managing infections in both human and animal populations [13]. The *nuc* gene, which encodes the thermonuclease enzyme, serves as a unique species-specific marker for *S. aureus*. Detection of the *nuc* gene using molecular techniques, such as polymerase chain reaction (PCR), is commonly used for the rapid and specific identification of *S. aureus* isolates from milk and dairy products [14]. *S. aureus* has the ability to develop resistance to a broad spectrum of antimicrobial agents. *S. aureus* has two mechanisms for resistance to β-lactam antibiotics. One is the production of β-lactamase enzymes, encoded by the *blaZ* gene, that hydrolytically degrade the core structure of β-lactam antibiotics such as penicillin and related antibiotics, rendering them ineffective [15, 16]. Detection of the *blaZ* gene in *S. aureus* strains isolated from dairy products indicate resistance to penicillin and related antibiotics, which complicate the treatment of infections caused by these strains [17]. The other is the expression of penicillin-binding protein (PBP 2a) encoded by the *mecA* gene, which confers resistance to β-lactam antibiotics, including methicillin and other β-lactam antibiotics [16, 18]. Methicillin-resistant *S. aureus* (MRSA) poses a significant risk to public health due to its resistance to multiple antibiotics, complicating treatment efforts [15, 16]. Detection of the *mecA* gene in *S. aureus* isolates from milk and milk products indicates potential public health risks associated with consuming contaminated dairy products. Globally, MRSA contamination in animal-derived foods, including milk and its products, is well-documented [2].

In Ethiopia, milk and milk products are distributed to consumers through both informal and formal marketing channels. In the formal system, milk is collected at dairy cooperatives or milk collection centers and then transported to processing plants. However, only a small portion, approximately 2% of the total milk produced, reaches the market through the formal dairy chain [19]. The majority of milk produced is consumed at home, processed into traditional dairy products, or marketed through informal marketing channels. The informal liquid milk market involves direct delivery of fresh milk by producers to local residents, restaurants, hotels, and informal vendors [20]. The informal market presents challenges related to quality control, hygiene, and product safety [19].

The Arsi highlands represent one of the major milk-producing areas in Ethiopia [21]. However, milk production in Ethiopia, including the Arsi highlands, often takes place under unhygienic conditions, increasing the risk of contamination by pathogens such as *S. aureus* [22]. Traditional production and handling methods along the milk value chain may lead to increased levels of microbial contamination [23], posing potential health risks to consumers. Common practices, such as consuming raw milk [22, 23], lack of pasteurization, utilization of nonfood-grade plastic containers, and inadequate refrigeration along the value chain, exacerbate these public health concerns [24]. Compounding these issues are reports of a high prevalence of *S. aureus* mastitis in the area, which can introduce the bacterium into milk [25, 26]. Despite these concerns, there is limited information regarding the occurrence and antimicrobial resistance of *S. aureus* in milk and milk products in the study area. To address this knowledge gap, a study was conducted to investigate the occurrence, antimicrobial susceptibility profiles, and the presence of resistance genes in *S. aureus* strains isolated from milk and milk products in the Arsi highlands of Ethiopia. Additionally, the enumeration of coagulase-positive staphylococci (CoPS) was carried out concurrently. The findings of this study can help in developing strategic interventions aimed at improving milk quality and ensuring its safety for consumers.

## Materials and methods

### Study area

**Fig. 1:**
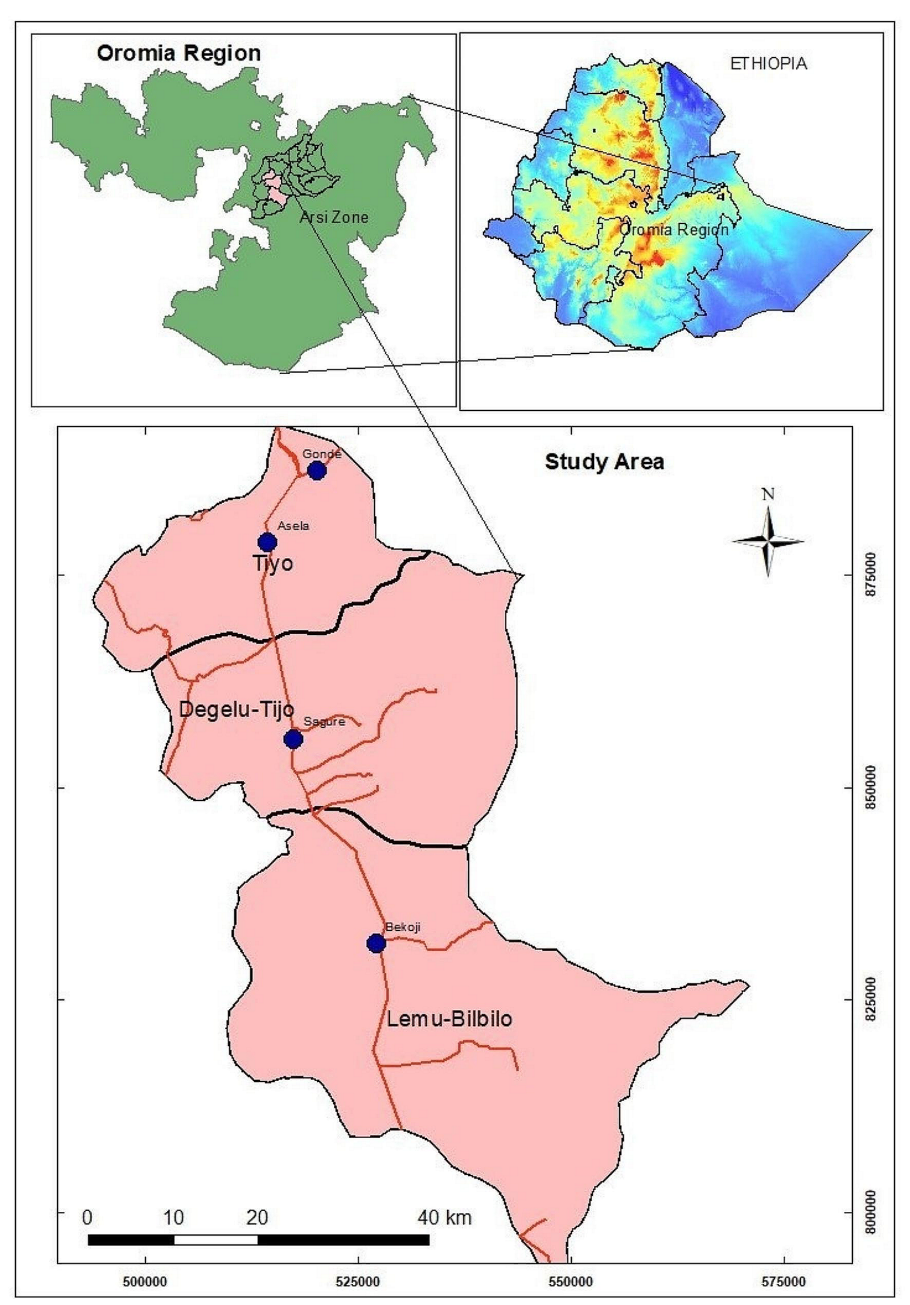
Map of the study areas

The study was conducted in the Tiyo, Digalu-Tijo, and Lemu-Bilbilo districts of the Arsi zone, Oromia Regional State, Ethiopia (Fig. 1). The respective administrative centers of these districts, namely Asella, Sagure, and Bokoji towns, and their surrounding areas were purposively selected based on milk production potential and accessibility. Asella is the capital of the zone, located in central Ethiopia, at a latitude and longitude of 7°57ˈN and 39°7ˈE, respectively. It is located 165 km southeast of Addis Ababa in the highland plateau region at an elevation of 2430 m above sea level. Bokoji is located 221 km southeast of Addis Ababa at an altitude and longitude of 7°35ˈN and 39°10ˈE, and an elevation of 2810 m above sea level. Sagure is located 189 km southeast of Addis Ababa at an altitude and longitude of 7°45ˈN and 39°9ˈE and an elevation of 2568 m above sea level. The study areas have a highland climate, with mean annual rainfall and average temperature of 1149 mm and 15.47 °C, respectively [27]. The area has a conducive climate for rearing specialized dairy breeds [21], and it was the area where the first small-scale dairy development was initiated in Ethiopia in collaboration with the Swedish government [28]. According to the Central Statistical Agency [29], the zone has a cattle population of 2,904,201, making it the largest among the zones in the Oromia region. Specifically, within this zone, there are 692,724 dairy cows, with 154,961 of them being crossbreeds. Livestock, particularly dairy production, plays a significant role in the livelihoods of farmers in these districts, where a mixed crop-livestock farming system is practiced. The dairy value chain in the Arsi zone involves various actors, including dairy producers, cooperatives, unions, collection centers, hotels/cafeterias, milk and milk product shops, and individual consumers. Recently, there has been a notable increase in the participation of women in the traditional street-side coffee business. They serve traditional coffee or coffee with milk on corridors of hotels/restaurants or in temporary tent-like coffee shops along the streets. Additionally, retail shops, primarily engaged in the sale of household goods, also sell milk.

### Study design and sample size determination

A cross-sectional study was conducted from March 2022 to February 2023 to determine the occurrence, antimicrobial resistance, and resistance genes of *S. aureus* in milk and milk products in the Arsi highlands of Oromia, Ethiopia. The sample size was determined using the method described by Thrusfield [30] for simple random sampling, with 95% confidence interval, 5% absolute precision, and an expected prevalence of 17% for *S. aureus* in milk and 14.29% in cottage cheese from a previous study [11]. Accordingly, the calculated minimum sample size was 217 for raw milk and 188 for cottage cheese. However, a total of 259 raw milk samples (96 from retail shops, 125 from traditional street-side coffee vendors, 25 from milk and milk product shops, and 13 from milk collection centers), and 219 samples of cottage cheese were collected randomly to increase precision. In addition, 25 yogurt samples were collected.

### Sampling methodology and sample collection

The study involved the collection of milk and milk product samples from various points along the dairy value chain, including milk collection centers (MCCs), retail shops, street-side traditional coffee vendors, milk and milk product shops, and open-air markets. Milk samples were collected from every other retail shops and road-side traditional coffee vendors located along the main routes. Due to limited number of MCCs and milk and milk product shops (commonly referred to as milk houses), milk and milk product samples were collected from all accessible establishments selling these items during the visit. Traditional yogurt samples were obtained from all available milk and milk product shops, while cottage cheese samples were randomly collected by sampling every other seller at open markets.

Approximately 20 mL of milk or yogurt, or 20 g of cottage cheese was collected into sterile screw-capped universal bottles. Prior to sampling, the containers were thoroughly agitated to ensure homogeneity. Strict aseptic procedures were followed during sample collection to prevent any potential contamination. Prior to sampling, participants were provided with a clear explanation of the study’s objectives, the data to be collected, and the strict confidentiality measures in place. Verbal consent was obtained before sample collection. The samples were then labeled and transported to the Asella Regional Veterinary Laboratory in an icebox for bacteriological analysis.

### Enumeration of coagulase-positive staphylococci

Sample preparation followed the International Organization for Standardization protocol [31], with minor modifications. For milk samples, serial dilutions were performed directly from the test sample. In the case of cottage cheese and yogurt (traditional fermented milk), serial dilution was performed from the initial suspension. An initial suspension (10^− 1^ dilution) was prepared by adding 10 mL of yogurt or 10 g cottage cheese into 90 mL of peptone water. Decimal dilutions up to 10^− 6^ were then prepared by transferring 1 mL of samples of the previous dilutions into 9 mL of peptone water, discarding 1 mL from the last dilution. The egg yolk was prepared by aseptically separating it from the egg white through multiple transfers between the shell halves. The separated yolk was then placed in a sterile flask, and four times its volume of sterile distilled water was added. The mixture was heated in a water bath at 47 °C for 2 h and then refrigerated at 4 °C for 24 h to allow the precipitate to form. To make the complete medium, the supernatant of the mixture and 3.5% potassium tellurite (Oxoid Ltd, Basingstoke, England) were added to Baird-Parker agar (BPA) (HiMedia, India) that had been boiled and kept at 47 °C. Subsequently, 100 µL of serially diluted milk or yogurt or cottage cheese samples were transferred from each dilution onto BPA supplemented with egg yolk and tellurite, and evenly spread by a bent glass rod. The plates were then incubated at 37 °C for 24–48 h. Black, shiny colonies surrounded by 2–5 mm clear zones were enumerated as coagulase-positive staphylococci (CoPS). The colonies on two consecutive plates containing 15–300 colonies were counted [32]. The CoPS count in the respective original sample was expressed as the number of colony-forming units per milliliter or gram (CFU/mL or CFU/g) of samples. The number *N* of identified CoPS present in the test sample was calculated using the following equation [33]:

$$N = \frac{{\sum C }}{{V\left( {{n_1} + 0.1{n_2}} \right)d}}$$

where $\sum C$ is the sum of the coagulase-positive staphylococcal colonies counted on the plates from two successive dilutions; *V* is the volume of inoculum placed on each plate, in milliliters; *n*_*1*_ is the number of plates selected at first dilution; *n*_*2*_ is the number of plates selected at the second dilution, and *d* is the dilution rate corresponding to the first dilution selected.

### Isolation and identification of *S. aureus*

*S. aureus* was isolated and identified from milk and milk products following the ISO 6888-1:1999 guidelines [32], with minor modifications. Briefly, 10 mL of milk or yogurt, or 10 g of cottage cheese was homogenized in 90 mL of sterile tryptone soya broth (HiMedia, India). For enrichment, the homogenate was incubated at 37 °C for 4–5 h [34]. A loopful of the culture was then plated onto BPA enriched with egg yolk and tellurite and incubated at 37 °C for 24–48 h. Two to five well-isolated typical colonies (black, shiny colonies surrounded by clear zones) were subcultured on nutrient agar (HiMedia, India) and incubated at 37 °C for 24 h. The isolates were further characterized based on colony morphology, Gram stain, mannitol fermentation, catalase test, and coagulase test. Slide coagulase test was performed using freeze-dried rabbit plasma (Santa Fe Drive, Lenexa, USA). Identified colonies were preserved in a 20% glycerol stock at -20 °C for further analysis.

### Antimicrobial susceptibility test

The antimicrobial susceptibility profiles of the isolates were determined phenotypically using the Kirby–Bauer disc diffusion method, following the guidelines established by Clinical and Laboratory Standards Institute (CLSI) [35]. Briefly, four or five pure, fresh overnight colonies from nutrient agar were transferred into a test tube containing 5 mL of sterile saline. The turbidity of the suspension was adjusted to a 0.5 McFarland standard. The inoculum was then evenly spread across the surface of a Mueller Hinton agar plate (HiMedia, India) using a sterile cotton swab. Excess fluid was removed by gently pressing the swab against the inner wall of the test tube. The plates were then allowed to air dry for 5 min at room temperature. Antimicrobial discs were carefully placed on the agar surface using sterile forceps and gently pressed down to ensure complete contact. The plates were left at room temperature for 5 min to facilitate drug diffusion, and then they were incubated at 37 °C for 16–18 h. The diameters of the inhibition zones were measured using a transparent ruler, and the *S. aureus* strains were categorized as susceptible, intermediate or resistant based on CLSI zone diameter breakpoints. The zone margin was defined as the area devoid of visible growth that could be seen with the naked eye [36]. *S. aureus* isolates were subjected to susceptibility testing against a panel of antibiotics (Oxoid, United Kingdom), including penicillin G (10 units), amoxicillin-clavulanic acid (10/20 µg), tetracycline (30 µg), streptomycin (10 µg), cefoxitin (30 µg), erythromycin (15 µg), ampicillin (10 µg), kanamycin (30 µg), trimethoprim-sulfamethoxazole (1.25/23.75 µg), chloramphenicol (30 µg), gentamicin (10 µg), and clindamycin (2 µg). The *S. aureus* ATCC25923 strain was used as a positive control.

### Detection of antimicrobial resistance genes and the *nuc* gene

DNA extraction from pure cultures of *S. aureus* was performed using the Qiagen DNeasy extraction kit (Qiagen, Germany), following the manufacturer’s instructions. The extracted DNA was then amplified using a 25 µL master mix consisting of 5 µL of PCR buffer, 0.8 µL of dNTPs (deoxynucleotide triphosphates), 2 µL each of forward and reverse primers, 0.5 µL of Taq polymerase enzyme, 12.7 µL of RNase-free water, and 2 µL of DNA template. PCR amplifications targeting *nuc*, *mecA*, and *blaZ* gene-specific fragments were conducted using thermal cycler (Biometra Gmbh, Germany) with the following cycling conditions: initial denaturation at 95 °C for 5 min, followed by 37 cycles of denaturation at 95 °C for 30 s, annealing at 55 °C for 30 s, and extension at 72 °C for 1 min; and a final extension at 72 °C for 10 min. The amplified products were separated on a 1.5% (w/v) agarose gel stained with ethidium bromide and visualized using UV illumination. The primer sequences and amplicon sizes for antimicrobial resistance genes and the *nuc* gene are presented in Table 1.

**Table 1:** Primer sequences and amplicon sizes for antimicrobial resistance genes and the *nuc* gene

Gene	Primer sequence (5’-3’)	Amplicon size	Reference
*Nuc*	F: GCGATTGATGGTGATACGGTT R: AGCCAAGCCTTGACGAACTAAAGC	279	[37]
*mecA*	F: GTAGAAATGACTGAACGTCCGATGA R: CCAATTCCACATTGTTTCGGTCTAA	310	[38, 39]
*blaZ*	F: TACAACTGTAATATCGGAGGG R: ATTACACTCTTGGCGGTTTC	861	[40]

### Data analysis

The data were entered into a Microsoft Excel spreadsheet and analyzed using STATA version 16 software (StataCorp, College Station, Texas, USA). Descriptive statistics were used to summarize the occurrence of *S. aureus* in milk and milk products, as well as the antimicrobial susceptibility profiles of the isolates. Logistic regression analysis was performed to assess the relationship between the presence of *S. aureus* and the type of sample or sampling location. CoPS count data were normalized by log_10_ transformation. One-way analysis of variance (ANOVA) was used to assess the association between CoPS count and the type of sample or sampling location. The geometric mean of the CoPS count of the samples was calculated. *P* ≤ 0.05 was considered statistically significant.

## Results

### Occurrence and concentration of *S. aureus* in milk and milk products

Of the 503 examined milk and milk product samples analyzed, 24.9% (CI: 21.1–28.6) tested positive for *S. aureus*. The occurrence was highest in raw milk (40.9%, CI: 34.9–47), followed by yogurt (20%, CI: 3.1–36.9) and cottage cheese (6.4%, CI: 3.1–9.7). These differences in occurrence among the sample types were statistically significant (*P* < 0.05). When comparing the likelihood of *S. aureus* occurrence among different sample types, it was observed that the probability of *S. aureus* contamination was 10.1 times higher in raw milk samples and 3.7 times higher in yogurt samples compared to cottage cheese (*P* < 0.05). *S. aureus* was detected in milk from MCCs, milk and milk product shops, retail shops, and traditional street-side coffee vendors, with occurrence proportions of 53.8, 44.8, 43.3, and 37.6%, respectively. However, no statistically significant difference in occurrence was observed among milk samples collected from different sampling locations. The occurrence of *S. aureus* in milk and milk products is presented in Table 2.

In our study, we observed variations in the concentration of *S. aureus* across different sampling locations. The geometric mean log_10_ CoPS was 5.3 log CFU/mL (2.2 × 10^5^) in milk collected from the bulk tank of MCCs, 5.1 log CFU/mL (1.11 × 10^5^) in milk and milk product shops, 4.3 log CFU/mL (1.9 × 10^4^) in traditional street-side coffees vendors, and 4.2 log CFU/mL (1.6 × 10^4^) in retail shops. The difference in mean log_10_ CoPS count among sampling locations was statistically significant (*P* < 0.05). The overall geometric mean of the CoPS count for raw milk collected from various sampling locations was 4.6 log CFU/mL (3.7 × 10^4^). Regarding milk products, the mean log_10_ CoPS in yogurt and cottage cheese were differed, with values of 3.8 log CFU/mL (5.57 × 10^3^) and 3.2 log CFU/g (1.59 × 10^3^), respectively. Importantly, we observed that *S. aureus* concentration was higher in raw milk compared to milk products (*P* < 0.05).

**Table 2:** Occurrence of *S. aureus* in milk and milk products

Type of sample	Sampling location	No. examined	No. positive	% positive (95% CI)	OR (95% CI)	*P*-value
Raw milk	Street-side traditional coffee vendors	125	47	37.6 (29–46.7)	-	-
	Retail shops	96	41	42.7 (32.7–53.2)	1.2 (0.7–2)	0.4
	Milk and milk product shops	25	11	44 (24.4–65.1)	1.3 (0.5–3)	0.6
	MCCs	13	7	53.8 (25.1–80.8)	1.9 (0.6–6)	0.3
Cottage cheese	Open markets	219	14	6.39 (3.1–9.7)	-	-
Yogurt	Milk and milk product shops	25	5	20 (3.1–36.9)	3.7 (1.2–11)	0.02
**Overall**		**503**	**125**	**24.9 (21.1**–**28.6)**		

*MCCs*, Milk collection center, *No* Number, *CI* Confidence interval, *OR* Odds ratio, *P* < 0.05

### Antimicrobial susceptibility profiles

The *S. aureus* isolates were tested for resistance against 12 different antimicrobials. The results of this part present antimicrobial susceptibility profiles of 39 randomly selected *S. aureus* isolates. The antimicrobial susceptibility profiles of *S. aureus* isolated from milk and milk products are summarized in Table 3. The isolates demonstrated the highest phenotypic resistance rates against ampicillin (89.7%) and penicillin G (87.2%). Tetracycline, amoxicillin-clavulanic acid, and cefoxitin displayed moderate resistance rates of 46.2, 28.2, and 25.6%, respectively. Notably, none of the isolates showed resistance to gentamicin, kanamycin, streptomycin, or chloramphenicol, while clindamycin, trimethoprim-sulfamethoxazole, and erythromycin showed low resistance rates. *S. aureus* is categorized as multidrug-resistant (MDR) when the isolate demonstrates non-susceptibility to at least one agent in three or more antimicrobial classes [41, 42]. In this study, 71.8% of the isolates exhibited multidrug resistance. Specifically, two isolates demonstrated resistance to seven classes of antimicrobials, while another two were resistant to six classes. Six isolates displayed resistance to five antimicrobial classes, and two isolates were resistant to four classes. Additionally, sixteen *S. aureus* isolates (41%) exhibited resistance to three classes of antimicrobials, while six were resistant to two antimicrobial classes. The two isolates that demonstrated resistance to seven antimicrobial classes were isolated from cottage cheese and milk sourced from a milk collection center. Moreover, the two isolates displaying resistance to six antimicrobial classes were identified in samples of cottage cheese.

**Table 3:** Antimicrobial susceptibility profiles of *S. aureus* isolated from milk and milk products (*N* = 39)

Class of antimicrobial agent	Antimicrobial agent	Susceptible n (%)	Intermediate n (%)	Resistant n (%)
β-lactams	Penicillin G	5 (12.8)	0	34 (87.2)
	Ampicillin	4 (10.3)	0	35 (89.7)
	Amoxicillin-clavulanic acid	28 (71.8)	0	11 (28.2)
Aminoglycosides	Gentamicin	37 (94.9)	2 (5.1)	0
	Kanamycin	31 (79.5)	8 (20.5)	0
	Streptomycin	38 (97.4)	1 (2.6)	0
Tetracyclines	Tetracycline	12 (30.8)	9 (23.1)	18 (46.2)
Sulfonamides	Trimethoprim-sulfamethoxazole	33 (84.6)	4 (10.3)	2 (5.1)
Cephalosporins	Cefoxitin	29 (74.4)	0	10 (25.6)
Phenicols	Chloramphenicol	39 (100)	0	0
Lincosamides	Clindamycin	31 (79.5)	4 (10.3)	4 (10.3)

*n*, number

### Detection of antimicrobial resistance genes and the *nuc* gene

**Fig. 2:**
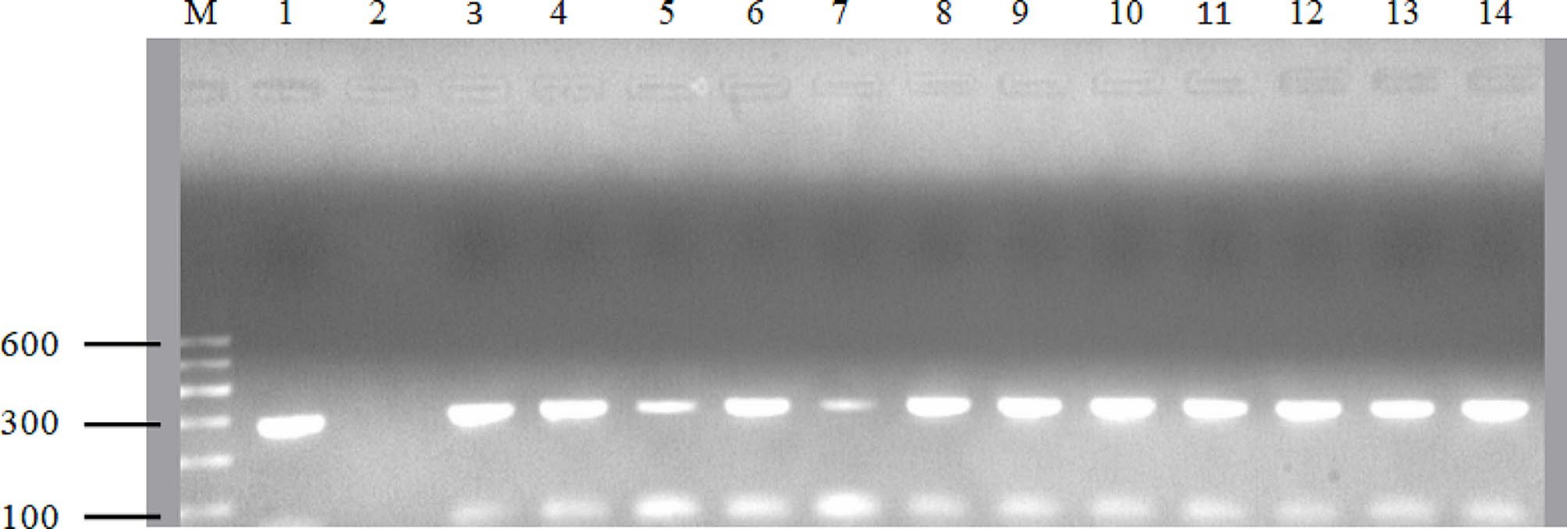
Agarose gel electrophoresis for the detection of the *nuc* gene (279 bp) in *S. aureus* isolates. Lane M, 100 bp DNA marker; Lane 1, positive control; Lane 2, negative control; Lane 3–14 positive for *S. aureus*

**Fig. 3:**
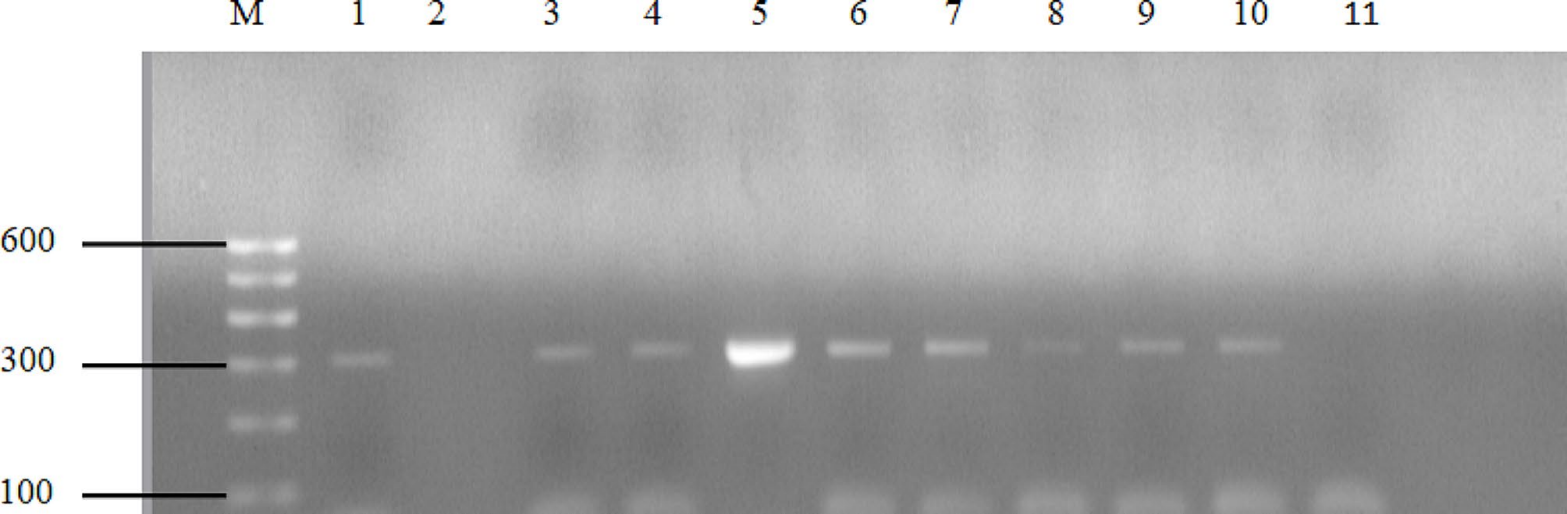
Agarose gel electrophoresis for the detection of the *mecA* gene (310 bp) in *S. aureus* isolates. Lane M, 100 bp DNA marker; Lane 1, positive control; Lane 2, negative control; Lanes 3–10, *mecA* gene positive isolates; Lane 11, negative isolate

**Fig. 4:**
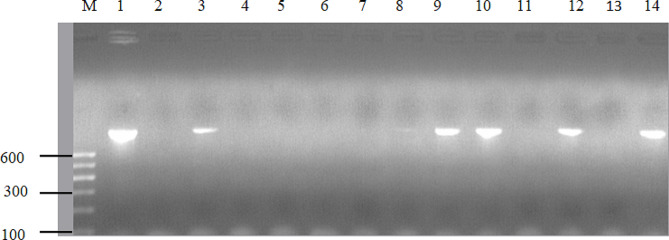
Agarose gel electrophoresis for the detection of the *blaz* gene (861 bp) in *S. aureus* isolates. Lane M, 100 bp DNA marker; Lane 1, positive control; Lane 2, negative control; Lanes 3, 9, 10, 12 and 14, positive for the *blaZ* gene; lanes 4–8, 11,13, negative isolates

In this study, sixteen isolates were further examined to determine the presence of specific genes, including *nuc*, *mecA*, and *blaZ* genes, using PCR. All the 16 isolates tested were confirmed to carry the *nuc* gene, with amplification products observed at approximately 279 bp. The *mecA* (MRSA) and *blaZ* genes were identified in 8 (50%) of these isolates each, producing bands at 310 bp and 861 bp, respectively. Of the eight MRSA (*mecA* positive) isolates, 3 (37.5%) were phenotypically MRSA (cefoxitin positive), while the *mecA* gene was detected in 5 (62.5%) isolates with a phenotypic MRSA-negative profile. All MRSA isolates were phenotypically resistant to penicillin, while 87.5% of MRSA isolates showed phenotypic resistance to ampicillin. Of the eight MRSA isolates, 4 (50%) displayed phenotypic resistance to tetracycline. Additionally, all isolates harboring the *mecA* gene exhibited multidrug resistance. All the eight isolates carrying the *blaZ* gene also exhibited phenotypic resistance to penicillin. Moreover, of the eight isolates carrying the *mecA* gene, four simultaneously harbored the *blaZ* gene. The PCR amplification of the *nuc*, *mecA*, and *blaZ* genes in *S. aureus* isolates is presented in Figs. 2 and 3, and 4.

## Discussions

*S. aureus* was identified in varying proportions across different sampling locations, with occurrence rates of 53.8, 44, 42.7, and 37.6% observed in milk samples collected from MCCs, milk and milk product shops, retail shops, and traditional street-side coffee vendors, respectively. However, the difference in occurrence among sampling locations was not statistically significant. The average prevalence across all sampling locations was calculated to be 40.9%. These findings closely align with a 40.6% occurrence reported in collection centers/cooperatives in Tigray, Ethiopia [43]. Consistent with the findings of the current study, a prevalence of 41.1% was reported in retail markets in Egypt [44], while a prevalence of 53% was reported in bulk cooling tanks in Portugal [45]. Similarly, in agreement with the present study, prevalence of 41.1% and 53% were reported in retail markets in Egypt [44] and bulk cooling tanks in Portugal, respectively. On the other hand, higher contamination rates of 80% and 72% were observed in MCCs in Sebeta [22] and Debre-Zeit [46] in Ethiopia, respectively, and 61.7% in retail raw milk samples in China [47]. In contrast, earlier investigations reported lower contamination rates, including 23.08% in Holeta at collection centers [11], 17.5% in Ambo and Bako towns in bulk tank milk at farm level [48] in Ethiopia, and 15.7% in raw cow milk in Iran [9]. A global meta-analysis documented a pooled prevalence of 33.5% in raw cow milk sampled from farms, retailers, and processing companies, which agrees with our findings [49]. In a recent meta-analysis, a pooled prevalence of 30.7% for *S. aureus* was reported in raw cow milk collected across milk value chain, from farms to processing plants, further supporting the finding of the present study [50]. The variations in occurrence among the different studies may be attributed to factors such as geographic location, hygiene practices, and differences in *S. aureus* isolation protocol. Our study utilized specific techniques such as sample enrichment and selective media, potentially contributing to a higher frequency of *S. aureus* isolation [51, 52]. The high occurrence of *S. aureus* in the present study indicates potential health risks associated with consumption of milk and milk products. This is particularly important in Ethiopia, where potential risky behaviors such as raw milk consumption are prevalent. The potential sources of contamination include udder diseases, unhygienic farm environments, and inadequate hygiene practices during milking, transportation, processing, storage, and distribution [9]. Thus, there is a need to improve hygiene standards and the health of dairy cows to ensure the safety of milk and its products. The provision of appropriate training for all actors across the dairy value chain could significantly mitigate contamination risks and reduce the incidence of foodborne illnesses [44].

In this study, 20% of yogurt samples tested positive for *S. aureus*, which is consistent with findings reported in other regions of Ethiopia. Prevalence of 17.5% in Addis Ababa [52], 24.14% in Holeta [11], and 25.5% in Tigray [43] have been documented. Conversely, other studies within the country have reported relatively lower prevalence, such as 3% in Jimma zone [53], 10.8% in Borena pastoral areas [23], and 13.11% in Ambo and Bako towns [48]. In Southwest Uganda, *S. aureus* was identified in 12.1% of fermented milk samples [54]. Our analysis of Ethiopian cottage cheese samples revealed a 6.4% occurrence of *S. aureus*, consistent with previous studies conducted in Ethiopia, which reported rates of 5% in Addis Ababa [52] and 7% in the Jimma zone [53]. Additionally, a prevalence of 10.9% was documented in Iran [9], further corroborating our findings. However, slightly higher prevalence rates were reported in other areas of the country: 18% in Jimma area [55], 18.03% in Ambo and Bako towns [47], and 28.6% in Tigray [43]. Similarly, a higher prevalence of 21.96% was reported in Turkey [56]. The variations in prevalence of *S. aureus* in yogurt and cottage cheese samples between our study and previous research may arise from differences in hygiene practices during preparation and handling. Contamination of yogurt and cottage cheese by *S. aureus* can originate from various sources, including unpasteurized milk, unclean equipment, and inadequate hygiene during handling. Therefore, proper sanitation and hygiene measures during the production and handling are crucial to prevent *S. aureus* contamination in yogurt and cottage cheese, thereby reducing the associated risks [24, 57].

In the current study, *S. aureus* contamination was observed to be higher in raw milk than in yogurt and cottage cheese. These findings are consistent with previous research conducted in Ethiopia [43, 48, 58] and Algeria [2], as well as with the result of a global systematic review and meta-analysis [59]. Additionally, our findings revealed that the mean log_10_ CoPS was greater in raw milk than in milk products. This variation may be linked to the traditional fermentation process employed during yogurt making or the heat treatments involved in cottage cheese preparation [2].

Our investigation revealed a higher contamination of *S. aureus* in yogurt samples compared to cottage cheese, in line with earlier findings in Ethiopia [60]. Moreover, the mean log_10_ CoPS was found to be greater in yogurt than in cottage cheese. The difference in contamination level may be attributed to the distinct characteristics of the two dairy products and their respective production processes. The liquid consistency of traditional yogurt provides a conducive environment for bacterial growth, facilitating easier multiplication compared to the denser and more compact structure of cottage cheese. On the other hand, cottage cheese undergoes processing steps such as curdling and draining, which are likely to contribute to the reduction in *S. aureus* presence. Moreover, the heating process involved in cottage cheese production has the potential to decrease the level of *S. aureus* contamination [24, 57].

In this study, the mean log_10_ CoPS for raw milk, yogurt, and cottage cheese were 4.6, 3.8, and 3.2 log_10_ CFU/mL, respectively. Similar findings were reported in previous studies conducted in Ethiopia [43, 61] and Tanzania [62] for milk and milk products. However, our results differed from previous studies that reported higher CoPS levels in raw milk in Ethiopia [11, 63] and Zimbabwe [61]. According to the European Commission’s microbiological criteria for dairy products, raw cow’s milk intended for direct human consumption is considered satisfactory if *S. aureus* counts in all samples does not exceed 500 CFU/mL. The result is considered unsatisfactory if the count in one or more samples is 2,000 CFU/mL or more. For counts between 500 and 2,000 CFU/mL, the result is considered acceptable if at least the counts in two of five samples does not exceed 500 CFU/mL [64]. Our study found that the mean CoPS for raw milk exceeded this this limit, suggesting potential health risks for consumers. In accordance with the East African Community (EAC) standards, the established maximum limit for *S. aureus* in both fermented milk and cheese is 100 CFU/g [65, 66]. Our findings reveal contamination levels exceeding these limits, highlighting potential public health risks, especially given that these products are consumed directly without additional treatment. The elevated CoPS levels in milk and milk products in the current study may be linked to suboptimal hygienic conditions during milking, transportation, processing, storage, and distribution. During our visits, we observed the absence of proper cooling facilities in most of milk and milk product selling establishments. These products were transported and stored at room temperature until sale, potentially leading to a gradual increase in bacterial counts over time [67]. Furthermore, higher CoPS counts may also be linked to delivery delays caused by long distances and transportation on foot [68]. It is generally considered that populations of enterotoxinogenic staphylococci between 10^5^ and 10^6^ CFU/g or mL or higher is required to produce detectable amounts of enterotoxin [69]. High concentration of *S. aureus* in milk and milk products can pose a significant risk to human health, as this bacterium can produce heat-stable toxins that can cause food poisoning if ingested [13].

In the current investigation, *S. aureus* demonstrated the highest resistance to ampicillin and penicillin G, with resistance rates of 89.7 and 87.2%, respectively. Similar resistance rates for penicillin, ranging from 69.9 to 98.5%, have been documented in various regions of Ethiopia [22, 70, 71], while rates of 76–91.3% have been reported in Algeria [2], Egypt [46], and China [5]. Consistent with our findings, previous studies have reported proportions of ampicillin-resistant *S. aureus* ranging from 70.9 to 98.5% in different regions of Ethiopia [11, 48, 53, 71], and rates ranging from 72.94 to 90.3% in Egypt [46], Turkey [56], Uganda [72], and China [5]. Supporting these observations, a global meta-analysis reported similarly high resistance levels, with penicillin resistance at 73.85% and ampicillin resistance at 59.63% [59]. The high levels of resistance displayed by *S. aureus* to ampicillin and penicillin pose a significant public health risk, emphasizing the need for stringent regulation in the administration of these drugs for animal infections. This high resistance observed in the current study is likely linked to the long-standing and indiscriminate use of these drugs in treating mastitis in dairy cows in Ethiopia [18, 73]. In Ethiopia, the practice of farmers self-prescribing veterinary drugs is widespread. With limited access to laboratory facilities, the treatment of animals often relies on symptom-based approaches rather than diagnostic testing. Furthermore, a lack of awareness about antimicrobial resistance and the consequences of inappropriate antibiotic use are prevalent among livestock owners in Ethiopia [73, 74]. The increased resistance observed in penicillin and ampicillin compared to other antibiotics may be attributed to *S. aureus* heightened susceptibility to β-lactam antibiotics resistance [18].

In our study, we found a tetracycline resistance rate of 46.2% among *S. aureus* strains. This pattern is likely influenced by the extensive use of oxytetracycline, the predominant antibiotic for treating animal bacterial infections in Ethiopia [73]. Our findings are consistent with previous studies documenting tetracycline resistance rates ranging from 32.4 to 47.8% in milk and milk products in Ethiopia [11, 53, 70], Algeria [2], Egypt [46], and China [5]. Contrary to our results, earlier studies in Ethiopia [22, 48], Iran [9], and Uganda [54] reported a high proportion of tetracycline-resistant *S. aureus* strains, ranging from 56.1 to 83.33%. The variation in tetracycline resistance levels between the current study and previous research likely arise from differences in antibiotic usage practices.

In this study, *S. aureus* demonstrated low resistance levels to clindamycin, trimethoprim-sulfamethoxazole, and erythromycin. Remarkably, none of the isolates displayed resistance to kanamycin, gentamicin, streptomycin, and chloramphenicol. Our findings are in line with previous research documenting a low prevalence of *S. aureus* resistance to these antibiotics [2, 5, 9, 11]. The observed low resistance levels may be linked to the limited use of these drugs in animal treatments in Ethiopia [70], suggesting a potential association between veterinary antibiotic usage and the development of antibiotic resistance [18].

In this study, 71.8% of the isolates exhibited multidrug resistance, defined as resistance to three or more antimicrobial classes, signaling a notable prevalence of antimicrobial resistance in the area. Our findings are consistent with elevated levels of MDR reported in other parts of the country, such as 65% in Holeta [11] and 61% in West Shewa [48]. Similarly, a study in Malaysia documented a comparable prevalence of multidrug resistance at 65.6% [75]. In contrast, lower levels of MDR were reported in other areas, including 43.9% in Ambo and Bako towns [48], 36.6% in Mukaturi and Sululta towns [76], 13.6% in Mekelle [70] in Ethiopia, as well as 12.8% in Iran [9]. The potentially alarming level of MDR identified in our study may be attributed to the irrational use of antibiotics in the treatment of animal infections in the country. Our results showed resistance to as many as seven classes of antimicrobials, raising significant public health concerns. The potential transmission of these resistant strains to humans through the handling and consumption of contaminated milk and milk products poses a clear threat. The presence of multidrug resistance complicates infection management, increases the risk of treatment failure, and can prolong hospital stays, leading to adverse patient outcomes and higher healthcare costs for both humans and animals [13].

In this study, all 16 *S. aureus* isolates subjected to PCR analysis were identified as *S. aureus*, confirmed by the presence of the *nuc* gene. The *mecA* and *blaZ* genes were identified in 8 (50%) of these isolates each. The identification of the *mecA* and *blaZ* genes highlights the presence of methicillin resistance and beta-lactamase production, respectively, which are critical mechanisms contributing to antibiotic resistance in *S. aureus* strains. Previous studies have reported varying prevalence of the *mecA* and *blaZ* genes in *S. aureus* strains isolated from milk and milk products across different regions: 4.1% in Algeria [2], 23.64% in India [77], 32.7% in Egypt [44], 42.6% in Brazil [78], and 51.6% in China [47] for the *mecA* gene; and 7.4% in Brazil [78], 25.8% in China [47], 84.9% in Egypt [44], and 92.73% in India [77] for the *blaZ* gene. The differences in *mecA* and *blaZ* gene prevalence observed between the current study and previous research may arise from various factors, including differences in antibiotic usage practices, genetic diversity among *S. aureus* strains, and differences in laboratory methodologies. In this study, out of the eight MRSA isolates confirmed based on the presence of the *mecA* gene, only 3 (37.5%) were phenotypically identified as MRSA through cefoxitin resistance. Surprisingly, the remaining 5 (62.5%) isolates, despite carrying the *mecA* gene, did not exhibit phenotypic resistance to cefoxitin. This discrepancy between genotypic and phenotypic results raises concerns about the reliability of cefoxitin resistance for MRSA identification. MRSA strains typically exhibit resistance to β-lactam antibiotics, including penicillin and ampicillin [16, 18], which was confirmed in our study. All MRSA (*mecA* positive) isolates were phenotypically resistant to penicillin, and a significant percentage (87.5%) of MRSA isolates also showed phenotypic resistance to ampicillin. In the present study, all the MRSA (*mecA* positive) isolates displayed multidrug resistance, confirming resistance of MRSA to multiple antimicrobial classes. This presence of multidrug-resistant *S. aureus* strains in milk and milk products, particularly those carrying the *mecA* gene, highlight the importance of antimicrobial stewardship programs aimed at promoting appropriate antibiotic prescribing practices and preventing the development of further resistance. All eight isolates carrying the *blaZ* gene displayed phenotypic resistance to penicillin, suggesting a strong correlation between the presence of the *blaZ* gene and resistance to penicillin among the tested isolates. The *blaZ* gene encodes for beta-lactamase enzymes, which are responsible for breaking down and deactivating beta-lactam antibiotics such as penicillin [16]. The elevated presence of *S. aureus* strains carrying *mecA* and *blaZ* genes in the present study raises significant health concerns regarding antibiotic resistance for consumers. Implementing effective preventive measures is crucial to mitigate these risks and safeguard consumer health.

## Conclusions

This study revealed the widespread distribution of *S. aureus* in milk and milk products in the Arsi highlands of Ethiopia. The isolates displayed high resistance to ampicillin and penicillin, with high level of MDR. Furthermore, our study found that the mean CoPS for milk and milk products exceeded international limits. These findings indicate potential health risks for consumers. The detection of the *mecA* (MRSA) and *blaZ* genes in selected isolates is of particular concern, posing a potential hazard to public health and presenting a serious challenge to antimicrobial therapy. This is particularly critical in Ethiopia, where the consumption of raw milk is widespread. The findings highlight the urgent need to improve hygiene standards in milk and milk product handling throughout the milk value chain. Additionally, stringent enforcement of regulations regarding antimicrobial usage in animal treatment is essential. Educational programs aimed at enhancing knowledge and raising awareness among farm workers, milk and milk product retailers, and milk collection center workers about the significance of good hygiene are essential. These measures are crucial in tackling the threats posed by *S. aureus*, including MRSA, and ensuring the safety of milk and its products for consumers.

### Electronic supplementary material

Below is the link to the electronic supplementary material.

Supplementary Material 1

## Data Availability

The datasets used and/or analyzed during this study are available from the corresponding author upon reasonable request.

## Abbreviations

CFU: Colony forming units
CoPS: Coagulase-positive staphylococci
ISO: International Organization for Standardization
CI: Confidence interval
OR: Odds ratio
MRSA: Methicillin-resistant *Staphylococcus aureus*
MDR: Multidrug resistance
MCC: Milk collection center
BPA: Baird-Parker agar
CLSI: Clinical and Laboratory Standards Institute
PCR: Polymerase chain reaction
EAC: East African Community
