# Supplementary material for: Occurrence, antimicrobial susceptibility, and resistance genes of Staphylococcus aureus in milk and milk products in the Arsi highlands of Ethiopia
Source: BMC Microbiol. 2024 Apr 16;24:127. doi: 10.1186/s12866-024-03288-3 (PMC11020821; doi:10.1186/s12866-024-03288-3)
Supplement: Supplementary file 1 — Supplementary Material 1 [file 12866_2024_3288_MOESM1_ESM.docx]

**Uncropped gels and blots**

1. ***Nuc* gene**


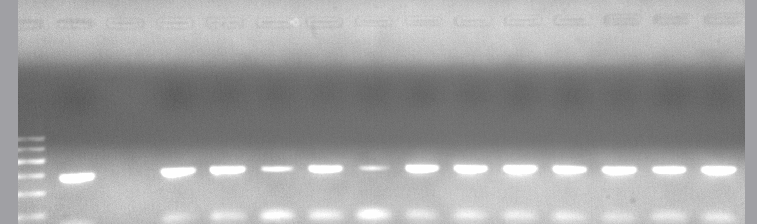


Agarose gel electrophoresis for the detection of the *nuc* gene (279 bp) in *S. aureus* isolates (**sample 1-12**). 100 bp DNA marker; Lane 1, positive control; Lane 2, negative control.


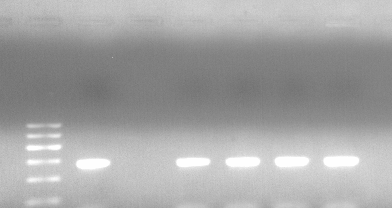


Agarose gel electrophoresis for the detection of the *nuc* gene (279 bp) in *S. aureus* isolates (**sample 13-16**). 100 bp DNA marker; Lane 1, positive control; Lane 2, negative control.

1. ***mecA* gene**


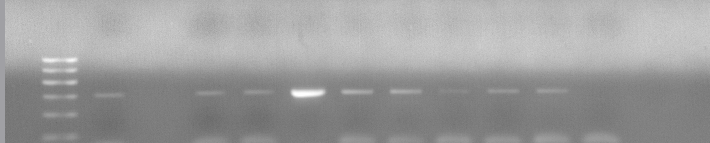


Agarose gel electrophoresis for the detection of the *mecA* gene (310 bp) in *S. aureus* isolates (**Sample 1-12**). 100 bp DNA marker; Lane 1, positive control; Lane 2, negative control

Note: **samples 13-16** are negative for *mecA* gene

1. ***blaZ* gene**


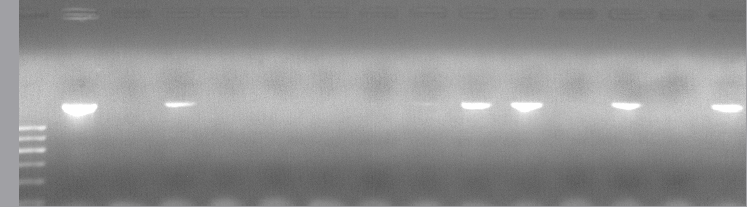


Agarose gel electrophoresis for the detection of the *blaz* gene (861 bp) in *S. aureus* isolates (**Samples 1-12**). 100 bp DNA marker; Lane 1, positive control; Lane 2, negative control


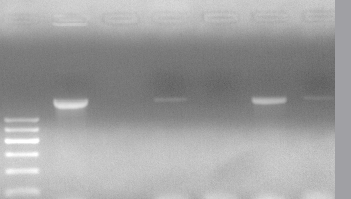


Agarose gel electrophoresis for the detection of the *blaz* gene (861 bp) in *S. aureus* isolates (**Samples 13-16**). 100 bp DNA marker; Lane 1, positive control; Lane 2, negative control

**Uncropped map of study area**

**
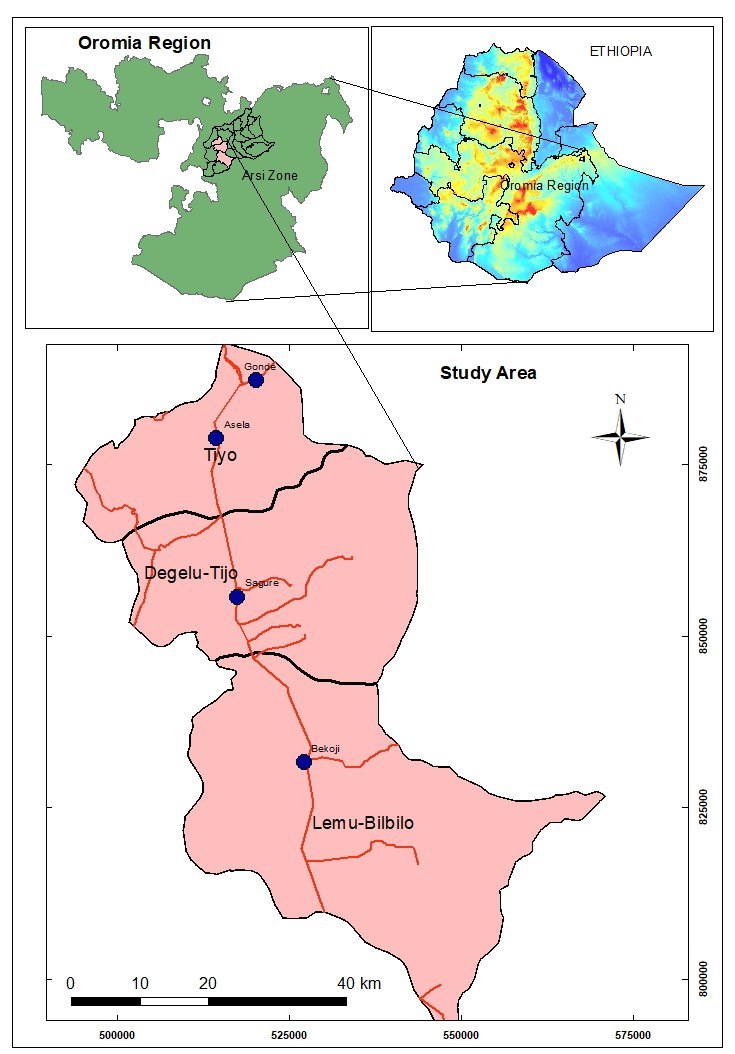
**
